# Altered Local Field Potential Relationship Between the Parafascicular Thalamic Nucleus and Dorsal Striatum in Hemiparkinsonian Rats

**DOI:** 10.1007/s12264-018-0312-9

**Published:** 2018-11-27

**Authors:** Haiyan Zhang, Jing Yang, Xuenan Wang, Xiaomeng Yao, Hongyu Han, Yunfeng Gao, Hongli Chang, Tianyu Xiang, Shuang Sun, Yanan Wang, Xiusong Wang, Min Wang

**Affiliations:** 1 Key Laboratory of Animal Resistance Biology of Shandong Province, College of Life Science, Shandong Normal University, Jinan, 250014 China; 2 School of Physics and Electronics, Shandong Normal University, Jinan, 250014 China

**Keywords:** Parkinson’s disease, Parafascicular thalamic nucleus, Dorsal striatum, Local field potential, Synchronization

## Abstract

The thalamostriatal pathway is implicated in Parkinson’s disease (PD); however, PD-related changes in the relationship between oscillatory activity in the centromedian-parafascicular complex (CM/Pf, or the Pf in rodents) and the dorsal striatum (DS) remain unclear. Therefore, we simultaneously recorded local field potentials (LFPs) in both the Pf and DS of hemiparkinsonian and control rats during epochs of rest or treadmill walking. The dopamine-lesioned rats showed increased LFP power in the beta band (12 Hz–35 Hz) in the Pf and DS during both epochs, but decreased LFP power in the delta (0.5 Hz–3 Hz) band in the Pf during rest epochs and in the DS during both epochs, compared to control rats. In addition, exaggerated low gamma (35 Hz–70 Hz) oscillations after dopamine loss were restricted to the Pf regardless of the behavioral state. Furthermore, enhanced synchronization of LFP oscillations was found between the Pf and DS after the dopamine lesion. Significant increases occurred in the mean coherence in both theta (3 Hz–7 Hz) and beta bands, and a significant increase was also noted in the phase coherence in the beta band between the Pf and DS during rest epochs. During the treadmill walking epochs, significant increases were found in both the alpha (7 Hz–12 Hz) and beta bands for two coherence measures. Collectively, dramatic changes in the relative LFP power and coherence in the thalamostriatal pathway may underlie the dysfunction of the basal ganglia-thalamocortical network circuits in PD, contributing to some of the motor and non-motor symptoms of the disease.

## Introduction

Parkinson’s disease (PD) is a complex neurodegenerative disorder with both non-motor and motor symptoms [1, 2]. The distinguishing pathological feature of PD is a progressive loss of dopamine in the nigrostriatal pathway [3, 4]. The dopamine deficiency results in abnormal neuronal activity and increased synchrony in the basal ganglia-thalamocortical circuits, which may be correlated with the motor symptoms [5]. Specifically, in the striatum, the main input structure of the basal ganglia, abnormal spiking activity and phase-locked spiking to cortical beta oscillations have been reported in PD patients and animal models of PD [6–8]. By contrast, few studies have explored PD-related changes in the oscillatory activity of local field potentials (LFPs) in the striatum. In akinetic mice, increased oscillatory activity in the delta and beta ranges is found in the dorsal striatum (DS) [9, 10]. In addition, one recent study suggests that the effects of dopamine depletion on LFP oscillations in the striatum may be task- and learning-dependent [11].

The centromedian-parafascicular complex (CM/Pf, or the parafascicular thalamic nucleus [Pf] in rodents), intralaminar thalamic nuclei, is the main glutamatergic input source of the striatum [12]. There is accumulating evidence that the CM/Pf is implicated in basal ganglia-related movement disorders and notably in PD [5]. It has been reported that CM/Pf neurons are lost in PD patients [13, 14] and 1-methyl-4-phenyl-1, 2, 3, 6-tetrahydropyridine (MPTP) neurotoxin-treated monkeys [15]. Alterations of firing rate and oscillatory activity in the CM/Pf have also been recorded in PD patients and anesthetized animal models of the disease [16–19]. Currently, only one study from our group has investigated these PD-related changes of neuronal activity in the CM/Pf in awake, behaving animals, revealing that these alterations are specifically related to ongoing activity during brain states (anesthetized *versus* awake state) and/or behavioral context (inattentive rest *vs* movement) [18, 20]. Therefore, exploring the effects of dopamine depletion on neuronal activity in the CM/Pf in different behavioral states remains of interest.

The thalamostriatal pathway has received increasing attention in PD research. For example, electrophysiological slice studies consistently have shown that the Pf–DS synaptic transmission is dysregulated after dopamine depletion [21–23]. However, PD-related changes in the relationship between neuronal activity in the Pf and DS have not yet been investigated. In addition, although the CM/Pf has been used as a target for deep brain stimulation (DBS) to treat some of the motor symptoms of PD [24–28], the underlying mechanisms are still unclear. CM/Pf DBS may exert its effects through the complex regulation of striatal neuronal activity and consequently influence some of the motor symptoms of PD. To provide an insight into this speculation, PD-related changes in the coherence between Pf and DS need to be considered.

Taking these findings together, this study aimed to investigate the effects of dopamine lesioning on LFP oscillations in the Pf and DS and importantly on their relationship in awake, behaving animals. Therefore, LFPs were simultaneously recorded from chronically implanted electrodes in both the Pf and DS during rest or treadmill walking in the dopamine-depleted hemisphere of hemiparkinsonian and neurologically intact rats.

## Materials and Methods

### Animals

Adult male Wistar rats (weighing 280 g−320 g) were obtained from the Animal Center of Shandong University one week before the experiments, and housed separately at constant room temperature (22 °C ± 2 °C) under a natural light-dark cycle (lights on from sunrise to sunset). With *ad libitum* access to water, food was limited to 10 g/day−20 g/day to keep animal weight constant. All animal care and experimental procedures were approved by the Animal Ethics Committee of Shandong Normal University, in accord with the NIH Guide for Care and Use of Laboratory Animals.

### Behavioral Training

Rats were trained to walk on a circular rolling treadmill for 30 min daily. A rotation speed of 12 r/min was used based on pilot experiments. Once rats could walk stably and continuously on the treadmill for 30 min, half received the dopamine lesion surgery described below. Five days after electrode implantation surgery, rats were re-trained on the treadmill until their performance recovered to the level before surgery. Finally, electrophysiological recordings were performed in all trained animals (dopamine-depleted and control animals) during periods of rest or treadmill walking.

### Unilateral Dopaminergic Cell Lesion and Assessment

A unilateral neurotoxic lesion of the substantia nigra pars compacta (SNpc) neurons was made using intracerebral infusion of 6-hydroxydopamine (6-OHDA) as we described previously [29]. Each rat was anaesthetized by intraperitoneal injection of 4% chloral hydrate (380 mg/kg), and then mounted on a stereotaxic instrument. 6-OHDA (4 μg/μL; Sigma, St. Louis, MO) dissolved in 0.9% NaCl with 0.02% ascorbic acid was injected through a glass micropipette at 0.5 μL/min into two target sites (1.5 μL each) in the medial forebrain bundle: −2.16 mm anteroposterior (AP), 2.1 mm mediolateral (ML), and 8.4 mm and 8.65 mm ventral from the skull surface according to the brain atlas of Paxinos and Watson’s [30]. After the infusion, the micropipette remained at each target site for 5 min to minimize diffusion of 6-OHDA. After 12 days of recovery, the rats were assessed for the severity of the dopamine lesion using the rotating behavioral response to apomorphine (0.5 mg/kg, intramuscular; Sigma). Only rats with > 210 contraversive rotations in 30 min after apomorphine injection were considered to have successful surgery and were used for the subsequent electrophysiological experiments.

### Electrode Implantation

Microelectrodes consisting of two independent bundles and two stainless steel wires as ground electrodes were implanted unilaterally in the Pf and DS. One group was implanted in the lesioned hemisphere. Each electrode bundle had 8 nickel-chromium Teflon-insulated microwires (25 μm diameter; California Fine Wire, Grover Beach, CA). The bundle for the Pf was divided into 2 wisps and arranged in a 1 × 2 matrix with ~ 100 μm between wisps, while the other for the DS had 4 wisps arranged in a 2 × 2 matrix with ~100 μm between wisps. Ground electrodes were in the corner of the array. Rats were anesthetized with 4% chloral hydrate (380 mg/kg, i.p. with an 80 mg/kg supplemental dose if necessary) and secured in the stereotaxic instrument. Electrodes were targeted over the Pf (−4.30 mm AP, −1.2 mm ML) and DS (−1 mm to 1 mm AP, −2.2 mm to −4 mm ML) and then were lowered gradually to their target depths (Pf and DS; 5.5 mm−6.5 mm and 4 mm−5 mm ventral from skull surface, respectively) and finally were fixed to the skull with dental cement and five screws. Ground wires were wrapped around three of the screws. After surgical recovery and acclimatization to the treadmill, electrophysiological recordings were carried out.

### Electrophysiological Data Acquisition

Using the 16-channel OmniPlex D Neural Data Acquisition System (Plexon Inc., Dallas, TX), LFP signals from the Pf and DS were recorded simultaneously for at least 30 min during periods of rest and treadmill walking. When recording during walking, two marks were manually added to the signal traces to indicate the times when the rat started to walk and stopped. Only signal segments between these marks were analyzed. The LFP signals were sampled at 1000 Hz with a band-pass filter set at 0.5 Hz−200 Hz; raw signal data were stored for later off-line analysis.

### Histology

**Fig. 1:**
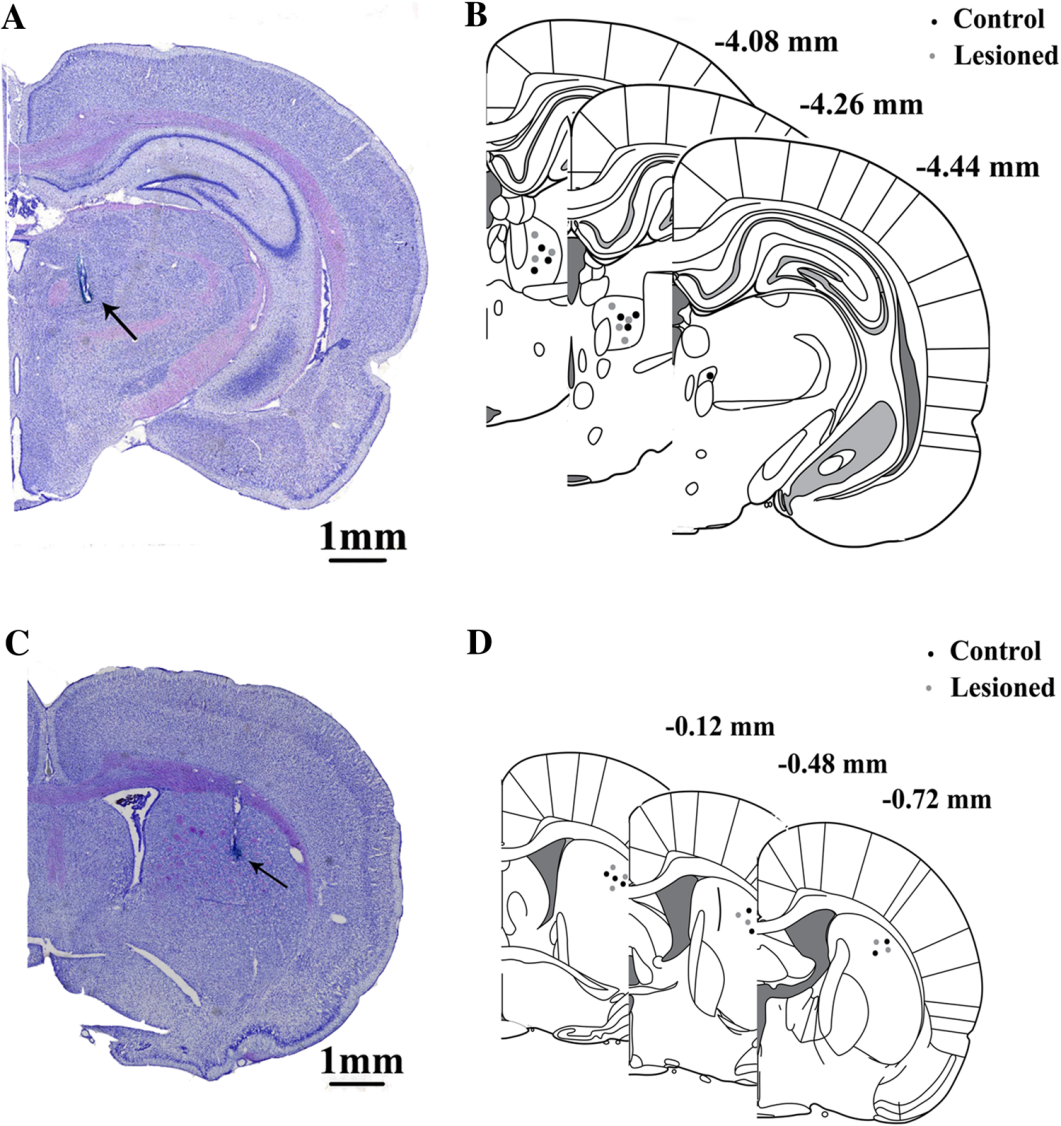
Verification of recording sites in the Pf and DS. **A**, **C** Representative images of cresyl violet-stained coronal sections through the Pf (**A**) and DS (**C**), showing the locations of the electrode tips (arrows). **B**, **D** Three schematics showing the sites of recording electrodes in the Pf (**B**) and DS (**D**) of both control (black dots, *n* = 7) and dopamine-lesioned rats (gray dots, *n* = 7). Numbers represent the distance from bregma in the anteroposterior plane. Pf: parafascicular thalamic nucleus; DS: dorsal striatum.

After completion of all experiments, rats were overdosed with 4% chloral hydrate (450 mg/kg) and a positive current of 10 μA was passed for 3 s × 10 times through the electrodes to mark the placement of the recording electrode tips. Rats were perfused intracardially with 200 mL saline followed by 200 mL 4% paraformaldehyde in phosphate buffer solution (PBS) containing 1% potassium ferricyanide. Then, the brains were removed and post-fixed in the same paraformaldehyde solution overnight at 4 °C, and then dehydrated in 30% sucrose in PBS for 72 h before they were sectioned at 40 μm in the coronal plane containing the Pf, DS, and SNpc. The SNpc sections were used to identify the lesions induced by 6-OHDA (data not shown). The detailed procedures and representative images of immunohistochemical staining were as described in our previous study [20]. The Pf and DS sections were mounted on glass slides, stained with cresyl violet, and visualized under a light microscope. Only rats with correct electrode placement in the Pf and DS were included for data analysis. The histological data from animals for further analysis are shown in Fig. 1.

### Data Analysis

LFPs were analyzed using NeuroExplorer version 5 (Nex Technologies, Littleton, MA) and LFP analysis software 2009 (http://www.nottingham.ac.uk/neuronal-networks/) in MatLab 2015a. Among the overall recording during rest and treadmill walking periods, 5 s × 10 representative epochs free of major artifacts were randomly selected for calculating LFP power and coherence. These data were from at least four microwires per brain region in each rat. To assess the LFP power change in a specific frequency band, the percentage of total LFP power (0.5 Hz–100 Hz) was calculated by fast Fourier transform (FFT) with a Hanning window (~1 Hz frequency resolution) in six frequency bands: δ (0.5 Hz–3 Hz), θ (3 Hz–7 Hz), α (7 Hz–12 Hz), β (12 Hz–35 Hz), low-γ (35 Hz–70 Hz), and high-γ (70 Hz–100 Hz). Averaged Welch plots were used to illustrate the power spectra across various frequencies. To visualize spectral power changes over time for the selected epochs, power spectrograms were constructed using continuous wavelet transforms.

To assess the alterations in the relationship between Pf and DS, both the mean coherence value and mean phase coherence were estimated in the 6 main frequency ranges. The mean coherence value provides a normalized frequency domain measure of the linear correlation between simultaneously-recorded LFP signals, ranging from 0 to 1, where 0 indicates no linear association and 1 indicates a perfect linear association. As a measure of the phase synchronization between two LFP signals, the phase coherence value was defined from 0 to 1, where high values indicate a high degree of phase synchronization and low values indicate unsynchronized signals [31]. Averaged frequency-coherence plots were used to visualize the changes in the relationship over frequency bands, while Rose plots illustrated the changes in phase coherence between the Pf and DS in a specific frequency band.

### Statistical Analyses

Data were analyzed with the statistical software package SPSS18 (SPSS Inc., Chicago, IL). All results are presented as mean ± SEM. The data for relative LFP power over frequency bands were analyzed using repeated-measures ANOVA with Greenhouse-Geisser correction, followed by *post hoc* Bonferroni tests, with lesion treatment as the between-subjects factor and frequency band as the within-subjects factor. The mean coherence and phase coherence measures in each single frequency band were analyzed by Student’s *t*-test. The level of significance was set at *P* < 0.05.

## Results

### Effect of Unilateral Dopaminergic Neuron Lesion on LFP Activity in the Pf

**Fig. 2:**
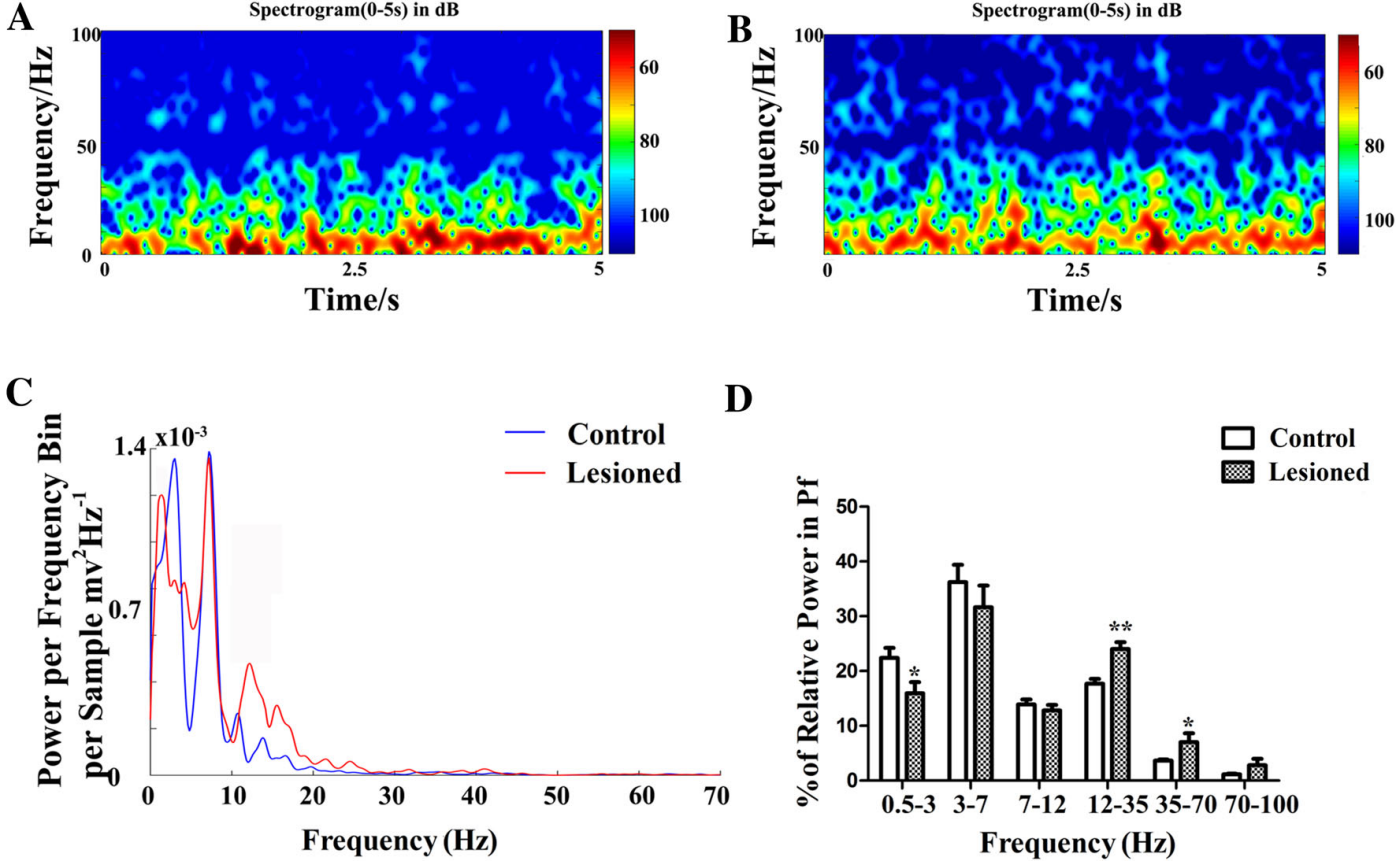
LFP activity in the Pf during rest episodes. **A**, **B** Representative scalograms showing the time-frequency plots of LFP power in control (**A**) and lesioned (**B**) rats. **C** Averaged Welch power spectra across frequency in the control and lesioned groups. **D** Mean relative LFP power in the Pf within the series of frequency ranges 0.5 Hz–3 Hz, 3 Hz–7 Hz, 7 Hz–12 Hz, 12 Hz–35 Hz, and 70 Hz–100 Hz in control (*n* = 7) and lesioned (*n* = 7) rats (*P < 0.05, **P < 0.001; Pf, parafascicular thalamic nucleus).

The scalograms showed a relatively distinct pattern of change of LFP power across time and frequency between the control (Fig. 2A) and lesioned (Fig. 2B) rats during the inattentive rest epochs. This result was supported by the FFT-based Welch analysis of LFP power spectra (Fig. 2C). To further evaluate and quantify the LFP power differences between the two groups, the relative powers in six frequency bands were compared. Repeated measures ANOVA with Greenhouse-Geisser correction showed a significant effect for the interaction between lesion and frequency band (*F*_(5, 60)_ = 3.66, *P* = 0.006, epsilon = 0.37). As shown in Fig. 2D, the lesioned rats had significantly higher LFP power not only in the β band (24.24 ± 1.09 *vs* 17.69 ± 0.89; *P* = 0.001), but also in the low γ band (6.92 ± 1.37 *vs* 3.64 ± 0.20; *P* = 0.035) than the control rats. However, the power was significantly lower in the δ band in the lesioned rats than in the control animals (16.04 ± 1.72 *vs* 22.39 ± 1.84; *P* = 0.027). There were no differences between the two groups in the other bands.

**Fig. 3:**
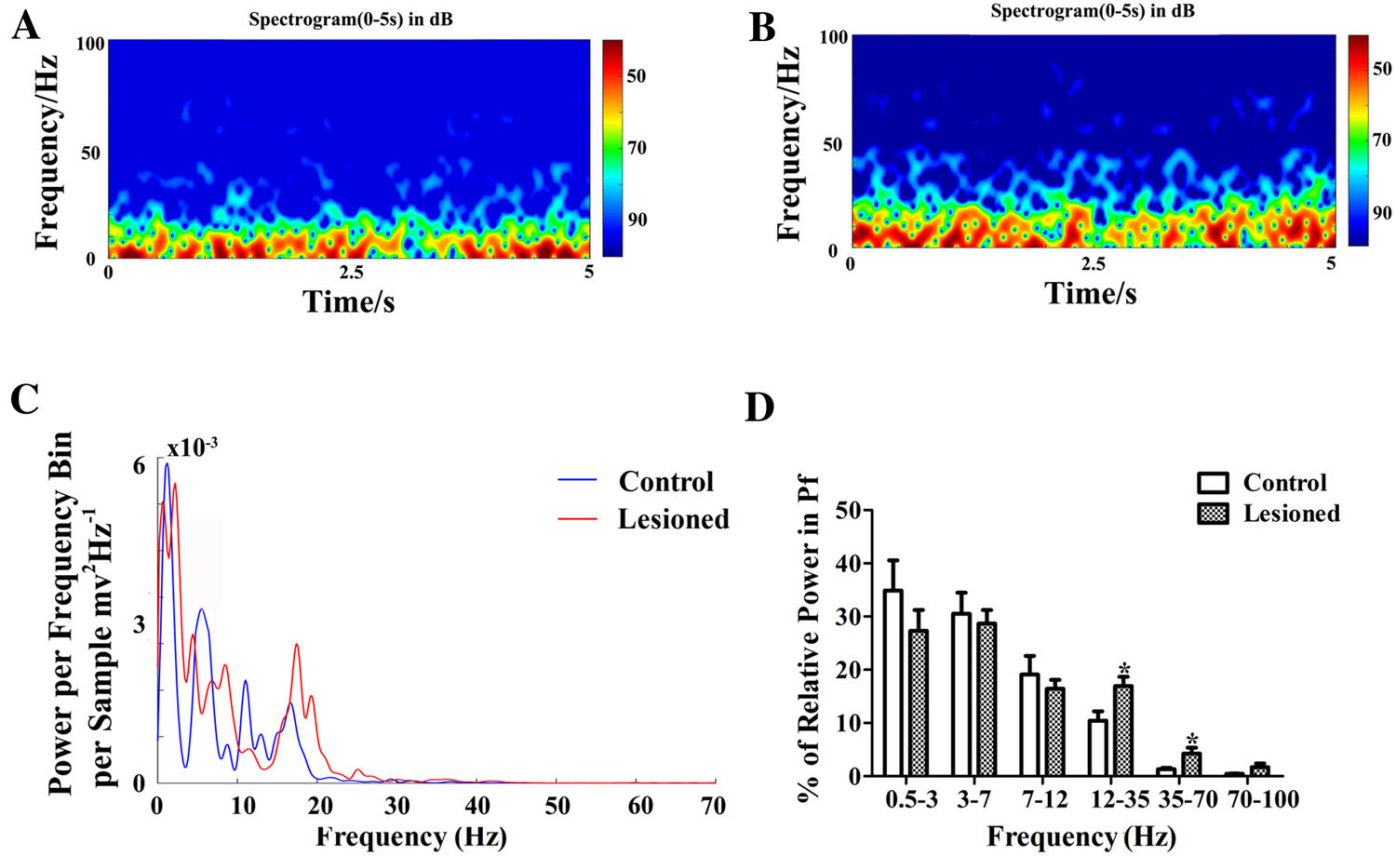
LFP activity in the Pf during treadmill walking epochs. **A**, **B** Representative scalograms showing the time-frequency plots of LFP power in control (**A**) and lesioned (**B**) rats. **C** Averaged Welch power spectra across frequency in control and lesioned groups. **D** Mean relative LFP power in the Pf in the frequency ranges 0.5 Hz–3 Hz, 3 Hz–7 Hz, 7 Hz–12 Hz, 12 Hz–35 Hz, and 70 Hz–100 Hz in control (*n* = 6) and lesioned (*n* = 7) rats (*P < 0.05; Pf, parafascicular thalamic nucleus).

During the treadmill walking epochs, there was a slight difference in the pattern of change in relative LFP power from that recorded during the rest epochs (Fig. 3A, C). Similarly, repeated measures ANOVA with Greenhouse-Geisser correction showed a significant effect for the interaction between lesion and frequency band (*F*_(4, 44)_ = 5.38, *P* = 0.001, epsilon = 0.39). The relative LFP power was also higher in both the β and low-γ bands in the lesioned rats than in the controls (β: 16.95 ± 1.74 *vs* 10.42 ± 1.75, *P* = 0.024; low-γ: 4.22 ± 1.14 *vs* 1.33 ± 0.24, *P* = 0.042). However, there were no significant changes in the δ or other bands. Interestingly, the relative LFP power in the lesioned rats was dramatically lower in all the low-frequency bands (from 0.5 Hz to 12 Hz) than in the control group (72.41 ± 3.90 *vs* 84.55 ± 3.15, *P* = 0.025). These results suggested that dopamine loss causes significant changes of LFP activity in the Pf in the lesioned hemisphere, confirming our previous findings [20].

### Effect of Unilateral Dopaminergic Neuron Lesion on LFP Activity in the DS

**Fig. 4:**
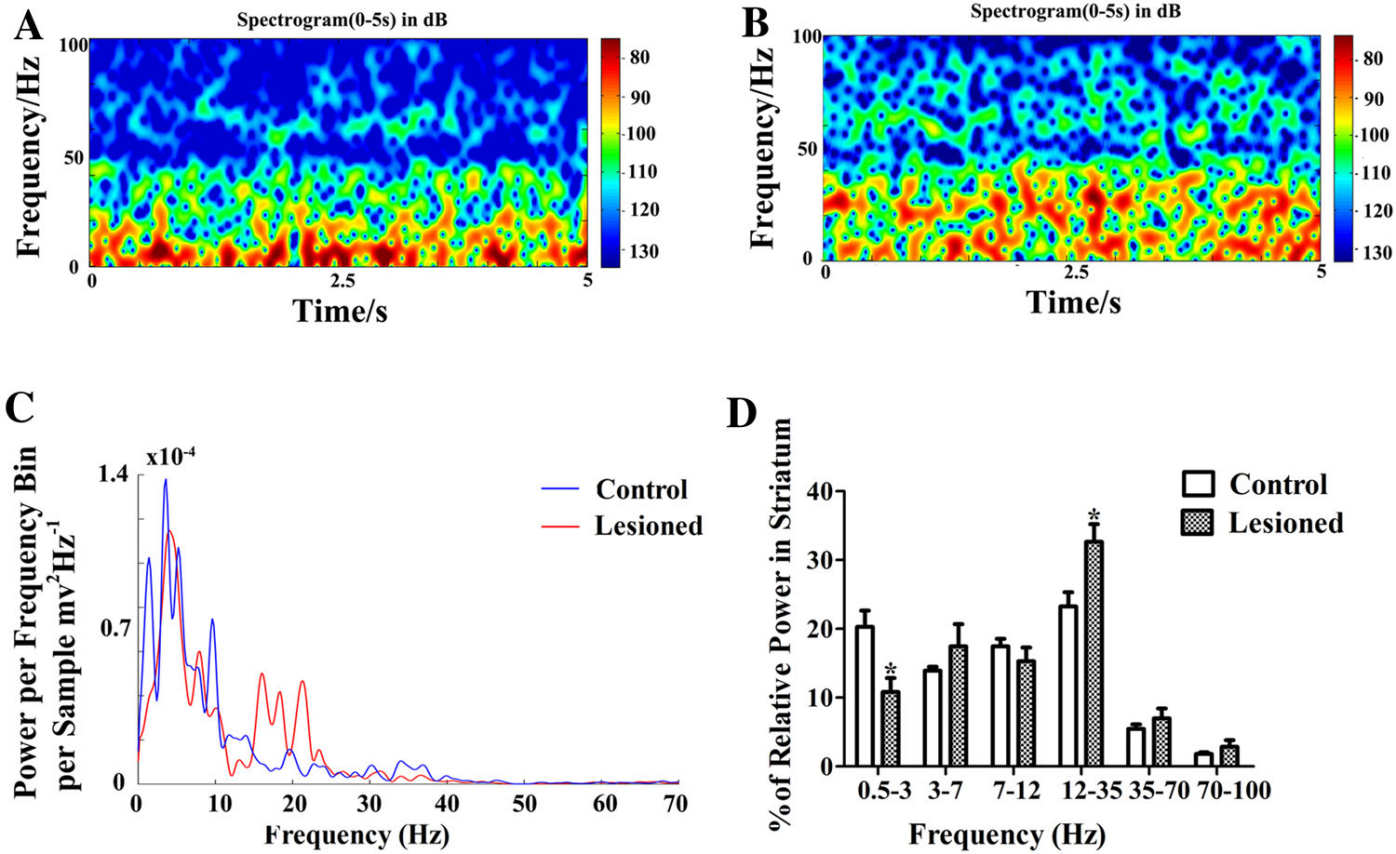
LFP activity in the DS during rest episodes. **A**, **B** Representative scalograms showing time-frequency plots of LFP power in control (**A**) and lesioned (**B**) rats. **C** Averaged Welch power spectra across frequency in both control and lesioned groups. **D** Mean relative LFP power in the DS in the series of frequency ranges 0.5 Hz–3 Hz, 3 Hz–7 Hz, 7 Hz–12 Hz, 12 Hz–35 Hz, and 70 Hz–100 Hz in control (*n* = 7) and lesioned (*n* = 7) rats (*P < 0.05).

**Fig. 5:**
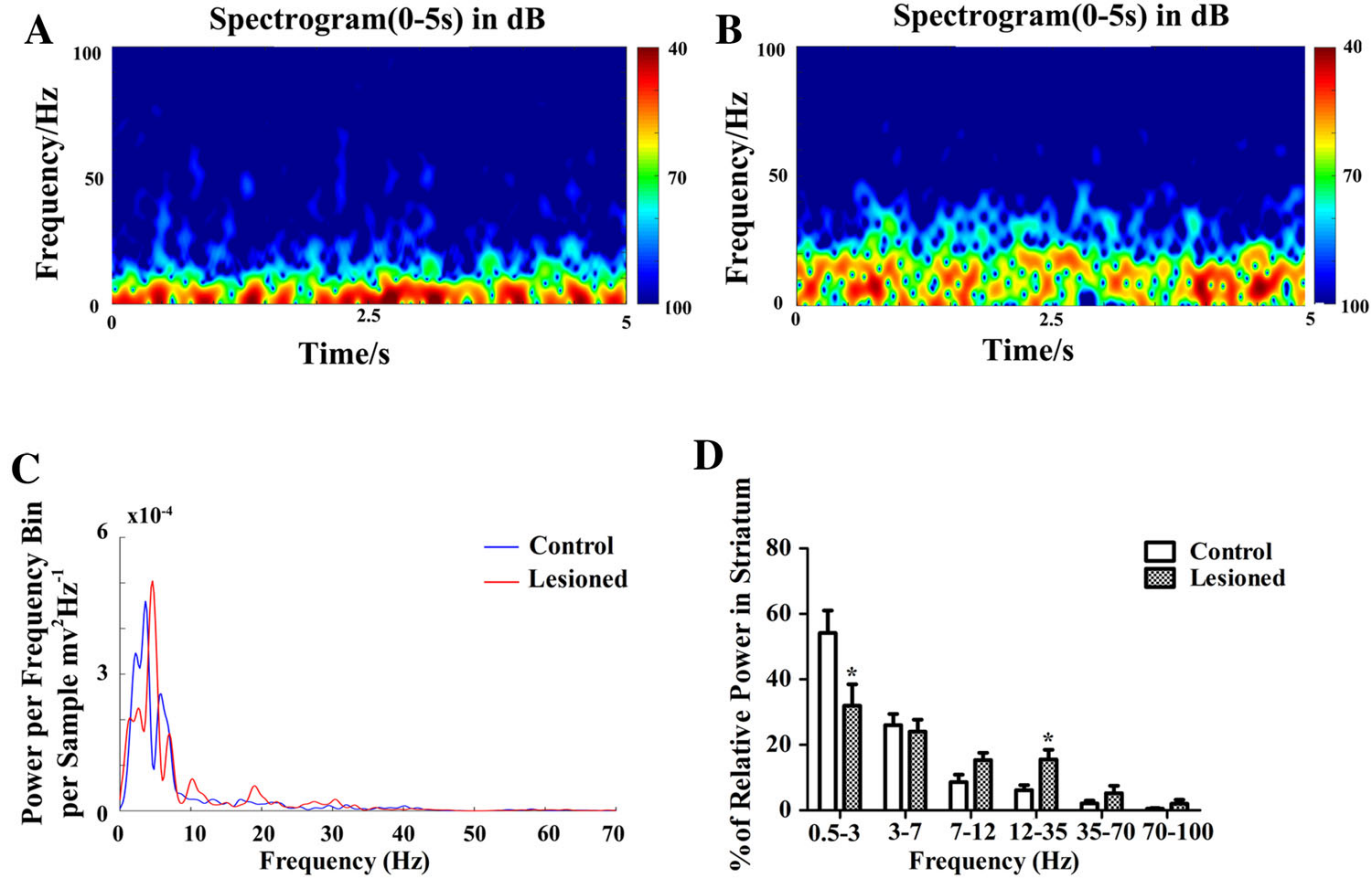
LFP activity in the DS during the treadmill walking epochs. **A**, **B** Representative scalograms showing time-frequency plots of LFP power in control (**A**) and lesioned (**B**) rats. **C** Averaged Welch power spectra across frequency in control and lesioned groups. **D** Mean relative LFP power in the DS in the series of frequency ranges 0.5 Hz–3 Hz, 3 Hz–7 Hz, 7 Hz–12 Hz, 12 Hz–35 Hz, and 70 Hz–100 Hz in control (*n* = 6) and lesioned (*n* = 7) rats (*P < 0.05).

To evaluate whether presumed changes in LFP activity in the DS were associated with the patterns of change in LFP power in the Pf, we analyzed LFPs recorded in the DS from the same animals. Visual inspection of representative scalograms (Figs. 4A, B and 5A, B) and Welch power spectral plots (Figs. 4C and 5C) showed that the dopaminergic neuron lesion led to evident changes in the DS LFP power, regardless of the state of the rats.

Similar to the results recorded in the Pf, repeated measures ANOVA with Greenhouse-Geisser correction showed significant effects for the interaction between lesion and frequency band during both epochs (rest: *F*_(6, 72)_ = 3.83, *P* = 0.002, epsilon = 0.35; treadmill walking: *F*_(5, 55)_ = 4.48, *P* = 0.034, epsilon = 0.32). The relative LFP power in the β band was strikingly higher in the dopamine-depleted rats during both the rest (32.66 ± 2.57 *vs* 23.26 ± 2.07; *P* = 0.015; Fig. 4D) and treadmill walking epochs (15.59 ± 2.89 *vs* 6.17 ± 1.56; *P* = 0.012; Fig. 5D). There were, nevertheless, significant decreases in the relative LFP power of the δ band in the lesioned rats during both the rest (10.82 ± 2.02 *vs* 20.29 ± 2.35; *P* = 0.01; Fig. 4D) and treadmill walking epochs (31.98 ± 6.54 *vs* 54.15 ± 6.88; *P* = 0.041; Fig. 5D). These results, along with other studies [8, 9, 32], indicate that an excessive β frequency LFP oscillation occurs in the DS in PD humans and animal models.

### Effect of Unilateral Dopaminergic Neuron Lesion on Pf–DS LFP Coherence

**Fig. 6:**
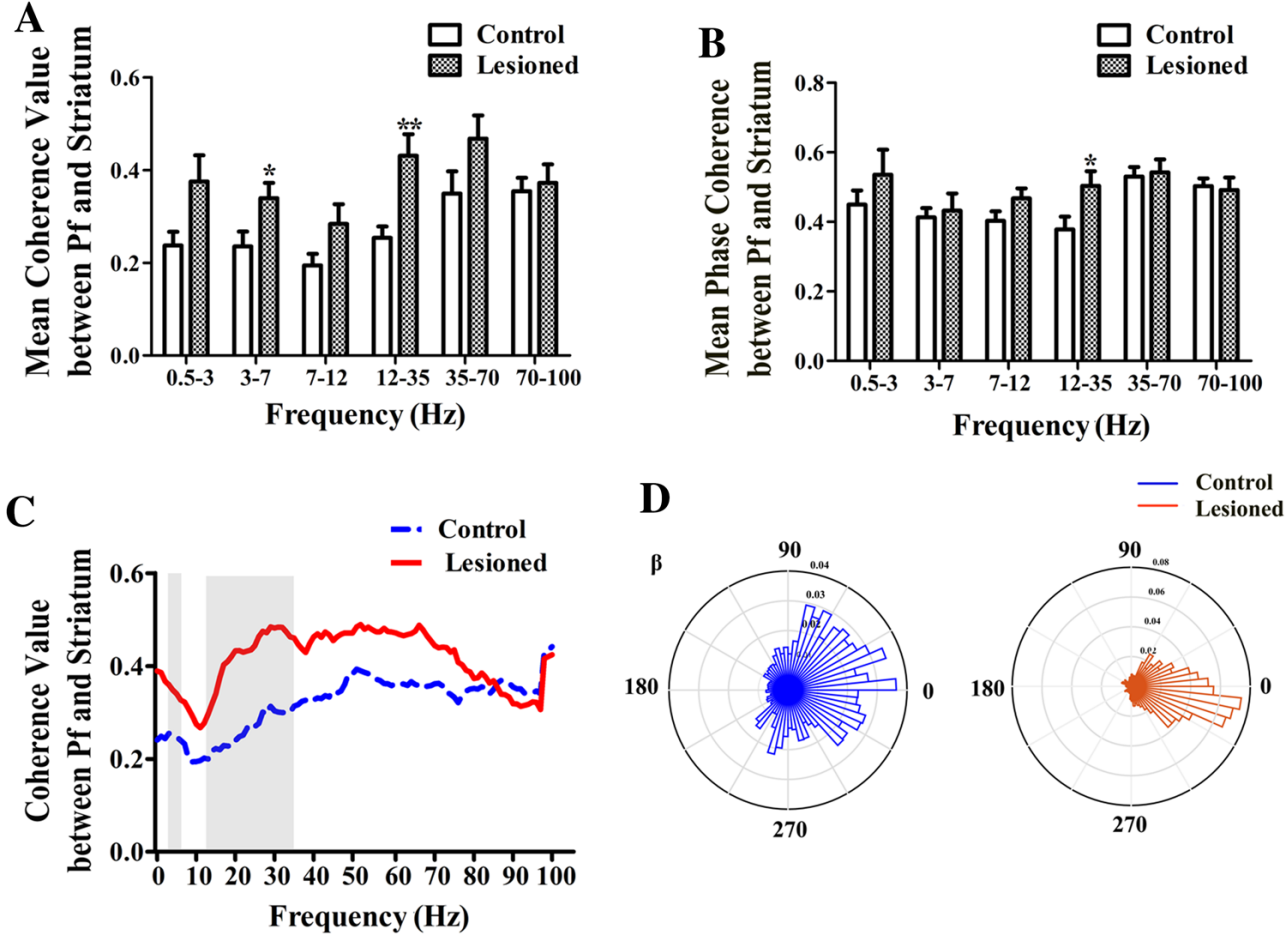
LFP coherence of Pf-DS during rest epochs. **A**, **C** Mean coherence values in the series of frequency bands 0.5 Hz–3 Hz, 3 Hz–7 Hz, 7 Hz–12 Hz, 12 Hz–35 Hz, 35 Hz–70 Hz, and 70 Hz–100 Hz (**A**) and averaged coherence values across frequency (**C**). **B**, **D** Mean phase coherence values in the series of frequency bands 0.5 Hz–3 Hz, 3 Hz–7 Hz, 7 Hz–12 Hz, 12 Hz–35 Hz, 35 Hz–70 Hz, and 70 Hz–100 Hz (**B**) and polar plots of phase coherence values in the (β) 12–35 Hz band (**D**) (*P < 0.05, **P < 0.001; Pf, parafascicular thalamic nucleus; DS, dorsal striatum).

**Fig. 7:**
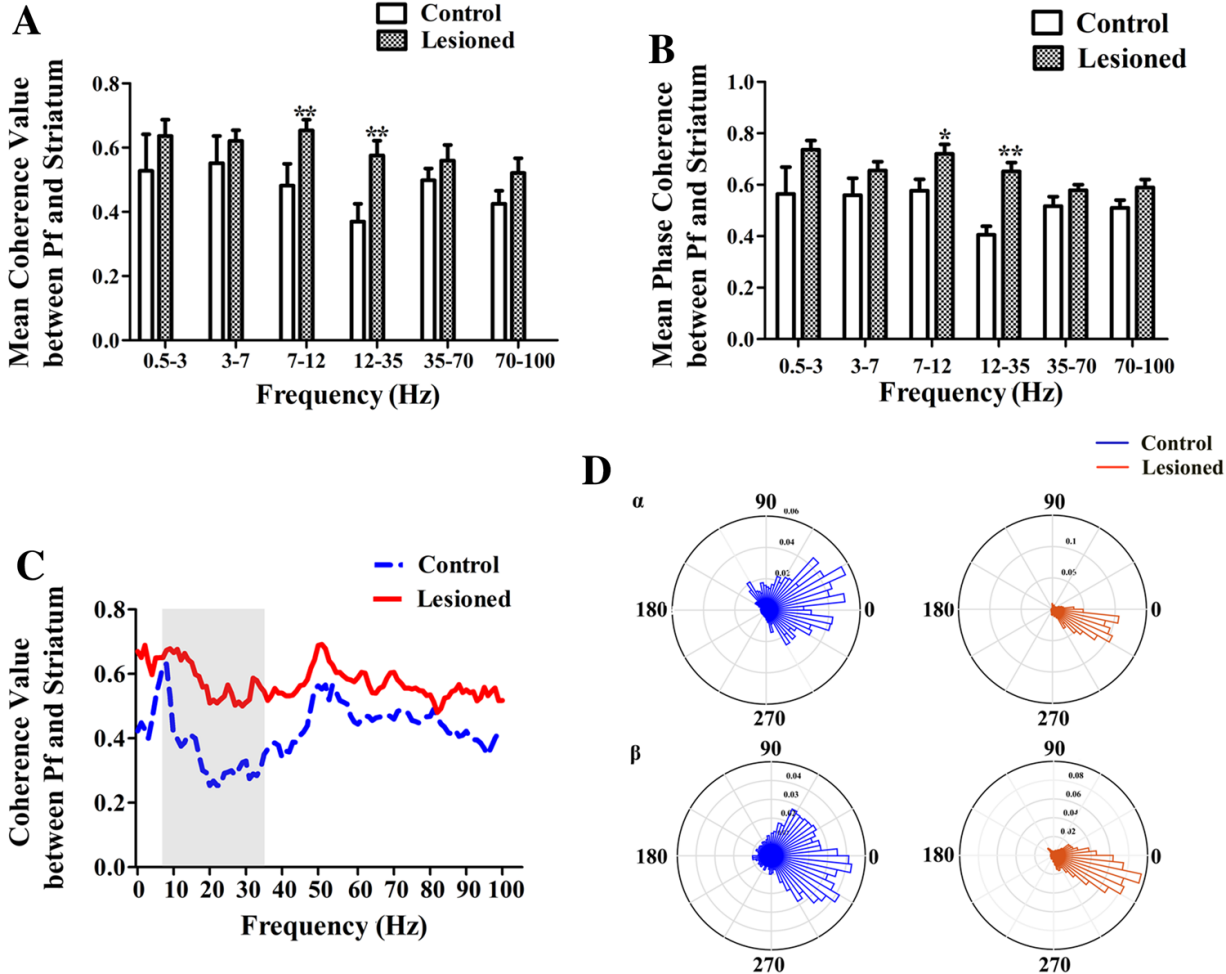
LFP coherence of Pf-DS during treadmill walking epochs. **A**, **C** Mean coherence values in the series of frequency bands 0.5 Hz–3 Hz, 3 Hz–7 Hz, 7 Hz–12 Hz, 12 Hz–35 Hz, 35 Hz–70 Hz, and 70 Hz–100 Hz (**A**) and averaged coherence values across frequency (**C**). **B**, **D** Mean phase coherence values in the series of frequency bands 0.5 Hz–3 Hz, 3 Hz–7 Hz, 7 Hz–12 Hz, 12 Hz–35 Hz, 35 Hz–70 Hz, and 70 Hz–100 Hz (**B**) and polar plots of phase coherence values in the (α) 7–12 Hz and (β) 12–35 Hz bands (*P < 0.05, **P < 0.001; Pf, parafascicular thalamic nucleus; DS, dorsal striatum).

To further evaluate the relationships between LFP oscillations in the Pf and DS after a dopaminergic neuron lesion, the mean coherence value and the phase coherence of the LFPs recorded in these structures during rest (Fig. 6) and treadmill walking (Fig. 7) were assessed.

In the analysis of mean coherence values during the rest epochs, there was an ensemble increase in the coherence between the Pf and DS in the lesioned rats (*n* = 7) compared to the controls, as well as significant increases in the θ and β bands (θ, 0.34 ± 0.03 *vs* 0.24 ± 0.03; *t*_(12)_ = −2.27, *P* = 0.043; β, 0.43 ± 0.05 *vs* 0.25 ± 0.02; *t*_(12)_ = −3.37, *P* = 0.006; Fig. 6A, C). The results of mean phase coherence showed a significant increase only in the β band in the lesioned rats (0.50 ± 0.04 *vs* 0.38 ± 0.04, *t*_(12)_ = −2.27, *P* = 0.043; Fig. 6B, D).

During the treadmill walking epochs, the mean coherence was significantly higher in the α and β bands in the lesioned rats than in the control rats (α, 0.65 ± 0.03 *vs* 0.43 ± 0.06, *t*_(11)_ = −3.47, *P* = 0.005; β, 0.58 ± 0.05 *vs* 0.32 ± 0.03, *t*_(11)_ = −4.44, *P* = 0.001; Fig. 7A, C). In the analysis of phase coherence, similar changes were found (Fig. 7B, D). Compared to the control rats, the lesioned rats showed higher phase coherence values in these two bands (α, 0.72 ± 0.04 *vs* 0.58 ± 0.04, *t*_(11)_ = −2.52, *P* = 0.028; β, 0.65 ± 0.04 *vs* 0.41 ± 0.03, *t*_(11)_ = −5.08, *P* < 0.001). These results suggested a prevalent increase in the synchronization of oscillatory activity between the Pf and DS in dopamine-depleted rats.

## Discussion

The aim of the present study was to explore alterations in neuronal oscillatory activity and its relationships in the thalamostriatal pathway after a dopamine lesion. We confirmed that dopamine loss induced increases in the relative LFP power in both the β and low-γ bands in the Pf during both states, but a decrease of power in the δ band only during rest state. In the DS, both an increase of power in the β band and a decrease of power in the δ band were found in the lesioned rats during both states. Importantly, we found synchronization of LFP oscillations between Pf and DS after a dopamine lesion. During the rest epochs, significant increases were found in both the θ and β bands for the mean coherence, but only in the β band for the phase coherence. During the treadmill walking epochs, significant increases were found in both the α and β bands for the two coherence measures.

It is well established that β-band oscillation of LFP in the basal ganglia is exaggerated in PD patients and animal models of the disease, and this is correlated with several motor impairments, such as bradykinesia and rigidity [5, 33, 34]. We also found enhanced LFP power in the β band in the Pf, along with augmented low-γ band oscillatory activity in the dopamine-lesioned rats during two states, confirming our previous findings [20]. However, only the power in the β band was notably increased during the movement state in the study of Wang *et al.* [20]. This inconsistency may be due to the different behavioral tasks used in the studies (food grabbing *vs* treadmill walking). Our results are also partially corroborated by other studies in PD patients and animal models [19, 35, 36]. Importantly, as the low-γ band oscillatory activity is correlated with resting tremor [36], increased power in this band in the Pf may partially account for the fact that lesions or high-frequency stimulation of the Pf can powerfully alleviate resting tremor [25, 27]. Taken together, these findings implicate Pf in the pathophysiology of PD, and especially in tremor at rest.

In the DS, we found enhanced LFP power in the β band after the dopamine lesion during the rest or treadmill walking state, consistent with previous studies [8, 9, 32]. However, we found a decrease of LFP power in the δ band in the dopamine-lesioned rats, which is not in line with previous research reporting increased power in this band [9]. This discrepancy may be attributable to the difference in animal models (dopamine transporter-knockout mice *vs* 6-OHDA-induced dopamine-lesioned rats) as well as the duration of the dopamine lesion (acute *vs* chronic). By contrast, Lemaire *et al.* found that LFP oscillations did not change in the pre-task baseline period, and their changes were task- and learning-related in the striatal dopamine-lesioned rats [11]. Therefore, changes in LFP oscillations after a dopamine lesion are correlated with both the extent of the dopamine lesion and the animal’s behavioral state; this remains to be further clarified.

In our study, LFP recording showed a strong correlation between the Pf and striatum and this relationship was exaggerated after dopamine loss. To our knowledge, this is the first study to report this result. The dopaminergic neuron lesion led to significant increases in the β and θ bands for the mean coherence, but only in the β band for phase coherence during the inattention rest periods. When in moving states, hemiparkinsonian rats showed increases in the α and β bands for two coherence measures compared to normal control rats. Increased synchronization between the Pf and striatum may be a result of the absence of dopamine in the basal ganglia and this makes the network more synchronized [37]. The projection and structure of thalamostriatal pathway dramatically change after dopamine depletion, such as the degeneration of CM/Pf cells [13–15] and a weakened Pf projection to the striatum in PD [21–23], which may be another reason for the electrophysiological changes in this pathway. In addition, high synchronization of oscillatory activity in the thalamostriatal pathway may contribute to the resonant activity throughout the basal ganglion-thalamocortical circuit [38]. Thus, excessive synchronous changes in the thalamostriatal pathway are not a causal but a central feature of PD [37].

DBS of the Pf can effectively reduce tremor and alleviate levodopa-induced dyskinesia in both PD patients and rodent models [24–28]. As previously noted, DBS-Pf may exert its effects by reducing not only the enhanced low-γ band oscillatory activity but also increasing the coherence between Pf and DS after adopamine lesion. Indeed, one recent study provided an insight into the latter possibility, showing that specific inhibition of Pf inputs to the DS reverses motor dysfunctions in dopamine-depleted mice [22]. However, little direct evidence has shown that these electrophysiological changes in/between the Pf and DS correlate with PD motor symptoms (e.g. tremor), although one study reported that in normal mice, increased β oscillations in the DS induced by optogenetic stimulation are accompanied by several parkinsonian-like motor deficits [39]. Therefore, further studies are needed to elucidate the mechanisms underlying DBS-Pf therapy in PD, and to clarify what the changes in LFP power in the Pf and DS as well as their synchronization mean in relation to PD-related motor impairments.

In conclusion, we found that loss of dopamine enhanced oscillations of LFP activity in both the Pf and DS, and importantly synchronization between the Pf and DS, especially in the β frequency range. Moreover, we found differential changes of LFP activity in the two areas and their synchronization in different behavioral states. Our study highlights the need for further investigation of these abnormalities as well as the thalamostriatal pathway in PD, in order to provide a better understanding of the neural mechanisms underlying the tremor in PD. Our findings, together with those of other studies [22], suggest that targeting the thalamostriatal circuit may be a new therapeutic approach for the treatment of some PD-related symptoms.
